# Potential Role of Exogenous Melatonin Supplement in Delirium Prevention in Critically Ill Patients: A Double-Blind Randomized Pilot Study

**Published:** 2018

**Authors:** Saeed Abbasi, Shadi Farsaei, Dorsa Ghasemi, Marjan Mansourian

**Affiliations:** a *Anesthesiology and Critical Care Research Center, Faculty of Medicine, Isfahan University of Medical Sciences, Isfahan, Iran. *; b *Department of Clinical Pharmacy and Pharmacy Practice, Isfahan Pharmaceutical Sciences Research Center, Faculty of Pharmacy, Isfahan University of Medical Sciences, Isfahan, Iran. *; c *Department of Clinical Pharmacy and Pharmacy Practice, Faculty of Pharmacy, Isfahan University of Medical Sciences, Isfahan, Iran. *; d *Department of Biostatistics, Faculty of Health, Isfahan University of Medical Sciences, Isfahan, Iran.*

**Keywords:** Melatonin, Delirium, Critical care, Clinical trial

## Abstract

Critically ill patients often suffer from disturbance of sleep-wake cycle and consequently delirium development, in intensive care units (ICU). In this study, we aimed to evaluate the effect of exogenous melatonin on delirium development and its related adverse sequelae in the subgroup of medical and surgical ICU patients. We performed a double-blind placebo-controlled randomized pilot study in adult patients admitted to the ICU. Recruited patients according to the considered inclusion criteria were randomized into treatment or placebo groups. Melatonin or placebo was administered in the first 24 h after admission, for 5 consecutive days. Incidence of delirium within 8 days of admission was reported as primary outcome in the different subgroups, and other pertinent clinical characteristics were evaluated as secondary outcomes. Out of the total of 172 patients assigned for the 2 study groups, 70 patients in placebo group and also 67 in melatonin group completed the study. We observed no therapeutic effect of melatonin on delirium prevention in ICU patients (percent of delirium in melatonin versus placebo group were 4.5% and 1.4% respectively). However, our findings indicated that melatonin might be more useful in preventing delirium development in medical ICU patients as compared to the surgical ICU patients. There were no intergroup differences in secondary outcomes with the follow-up ending on May 2016. Our findings suggested melatonin might be a potential option for prevention of delirium in medical ICU patients.

## Introduction

Delirium is characterized by acute central neural system disorder, ranging from 11% to 80% in intensive care units (ICU) (1). It is a neuropsychiatric syndrome with disturbances in consciousness, attention, cognition, and perception. This syndrome has an acute onset and a fluctuating course that is usually developed within hours and continued from several days to several weeks, or even months (2, 3).

Pathophysiology of delirium is likely due to reversible impairment of cerebral oxidative metabolism and abnormalities of several neurotransmitter systems, such as dopamine (4).

Delirium is associated with higher mortality rates, especially in ICU patients (mortality risk ratio in critically ill patients was 2.19) (5, 6). In addition, delirium development in ICU patients is associated with other complications such as poorer functional outcome, increased length of hospitalization, and higher rate of long-term cognitive impairment after hospital discharge (2, 3).

Delirium could be evaluated through reliable tools, mostly known as “Confusion Assessment Method for the Intensive Care Unit (CAM-ICU)” and “Intensive Care Delirium Screening Checklist (ICDSC)”. CAM-ICU indicated a high sensitivity (93%) and specificity (89%) for the diagnosis of delirium, while the ICDSC in ICU patients showed a higher sensitivity as compared with the CAM-ICU (99%) but a lower specificity (64%) (7, 8).

Various predisposing factors contributed to delirium development especially in the critically ill patients (9, 10). Accordingly multicomponent, non-pharmacological intervention strategies including minimization of delirium risk factors are effective in non-ICU patients, but they may be less likely to prevent delirium in ICU patients (10).

Haloperidol has been traditionally used as the drug of choice for prevention and treatment of ICU delirium (11). Recent studies also revealed that second-generation antipsychotics such as quetiapine, risperidone, and olanzapine have a beneficial role in prevention and treatment of ICU delirium (10, 12). However, adverse reactions limit using of haloperidol or other antipsychotics therapy (3, 13).

Therefore, High incidence of delirium and its related adverse sequelae in one hand, and reported adverse reactions of traditional therapies on the other hand, necessitate new approaches in prevention and treatment of delirium. Although changes in melatonin concentration that influences circadian rhythm may play an important role in developing delirium, few studies exist that consider the effects of melatonin supplementation for prevention of delirium (3, 14).

However, to the best of our knowledge, not only there is not any study conducted to evaluate the role of melatonin in prevention and treatment of delirium in ICU patients, but also there is controversy about the beneficial role of that in other studies (1, 15). Therefore, the primary objective of this study was to evaluate the effects of melatonin on the frequency of delirium among ICU patients. We also considered duration of ICU and hospital stay, length of delirium, the rate of cumulative dose of prescribed haloperidol (the sum of mg doses of haloperidol which was prescribed to control delirium), and mortality rate during hospitalization, as the secondary outcomes in our study. 

## Experimental

We conducted a double-blind placebo controlled randomized clinical trial to evaluate the efficacy of melatonin supplements compared to the placebo for delirium prevention in ICU patients. 

This study was approved by research ethics committee of Isfahan University of Medical Sciences and it was performed in accordance with the Declaration of Helsinki and subsequent revisions (16). This trial has also been registered in Iranian registry of clinical trials (IRCT: 2015022421159N1).

Patients were eligible for inclusion, if they met all of the following criteria:

Identified within the first 24 h of admission in the ICU

Age >18

Healthy gastrointestinal function (patients tolerated oral medications by gavage or mouth)

Richmond Agitation Sedation Scale (RASS) >-4 

Glasgow coma score (GCS) >8 

No basic delirium or mood changes before admission in ICU

The patients were excluded from the study, if they had less than 5 days ICU stay, sensitivity reaction to the melatonin supplement, pregnancy, previous history of seizure, or severe heart failure (New York Heart Association (NYHA) classification III/IV). Recruited patients were allocated to one of the two groups (melatonin and placebo) using a computerized random number generator. Placebo was prepared in the pharmaceutical laboratory of the pharmacy faculty of Isfahan University of Medical Sciences which is similar in size and color with melatonin tablet.

The medications were administered in a double blind manner, so different codes were allocated to the container of at least 5 tablets (melatonin or placebo) and then random assignment of patients to different codes was carried out. The codes that identified study group, was kept confidential by a third party and neither the investigator or ICU staff nor the patients were aware of treatment allocation until the study was over.

After the written consent was obtained from the patients or their proxy decision-makers, placebo or melatonin (3 mg) tablet was administered in the first 24 h at 9:00 p.m. and continued for 5 continuous days. They were followed daily for additional 3 days (minimum duration of follow-up was 8 days) to assess the frequency of delirium and related clinical parameters. Only the follow-up was continued for the patients staying in ICU, who developed delirium on the eighth day. 

The frequency of delirium as the primary outcome was tested daily using validated Persian version of standard CAM-ICU tools (17, 18). However, there were some pros and cons from both CAM-ICU and ICDC methods for delirium assessment. Nevertheless, CAM-ICU is more frequent for ICU patients and has been verified in various languages throughout the world (7, 8). This test provides high sensitivity, specificity, and inter-observatory reliable tools for rating the incidence of delirium.

CAM-ICU demonstrates four key features, which include: 1) An acute onset of mental status change or a fluctuating course, 2) Inattention, 3) Disorganized thinking, and 4) Altered level of consciousness. Accordingly, the patients were considered positive for delirium, when features 1 and 2, and either the third or fourth features were present (8). Therefore, each item was scored according to the response of the patients to each question asked during the standardized interview.

We also recorded baseline characteristics (age, sex, reason of ICU admission, documented past medical history of hypertension, diabetes mellitus and cardiovascular diseases, chronic organ insufficiency, history of previous cigarette smoking and acute physiology and chronic health evaluation (APACHE II) score) on admission. Information of patient isolation during ICU stay was also gathered during follow-up period. In addition, sequential organ failure assessment (SOFA) score and the chance of developing delirium, as assessed by PRE-DELIRIC model (PREdiction of DELIRium in ICu patients) were calculated in daily basis in our study. 

PRE-DELIRIC model is a reliable tool administered to forecast the delirium chance in each patient by using ten risk factors including age, diagnosis, urgent admission, morphine use, infection, coma, use of sedative, level of urea, metabolic acidosis, and APACHE II score (19, 20).

Moreover, duration of delirium, cumulative dose of prescribed haloperidol, period of ICU and hospital stay, and mortality rate were considered as the secondary outcomes in our study, and the related data were gathered from medical charts, nurses, or care providers. 

A research assistant was trained under didactic sessions by the expert clinicians, and tested to assure the reliability of the measured outcomes. All the gathered information related to patients were kept confidential, and all patients received routine ICU care to prevent delirium, including pain and sedative management, improving sleep period, and early ICU mobilization. Moreover, the patients received standard care, in case delirium had been occurred. 

*Statistical analysis*

All the collected data were entered in standard format in to SPSS (Statistical Package for the Social Sciences) ver. 18 software, for further analysis.

Frequencies, and percentages, were used for categorical variables, and mean and SD were used for continuous variables. Moreover, chi-square and independent sample *t*-test were used to compare categorical and continuous variables between the groups, respectively. 

Binary logistic regressions were used to analyze the relationship between melatonin usage, frequency of delirium and mortality rate by considering possible covariates. The applied simple linear regression showed possible relationship between melatonin usage and delirium duration, cumulative dose of prescribed haloperidol and duration of ICU and hospital stay. All statistical analyses were conducted at a significant level (*P-*value ≤ 0.05).

## Results

**Figure 1:**
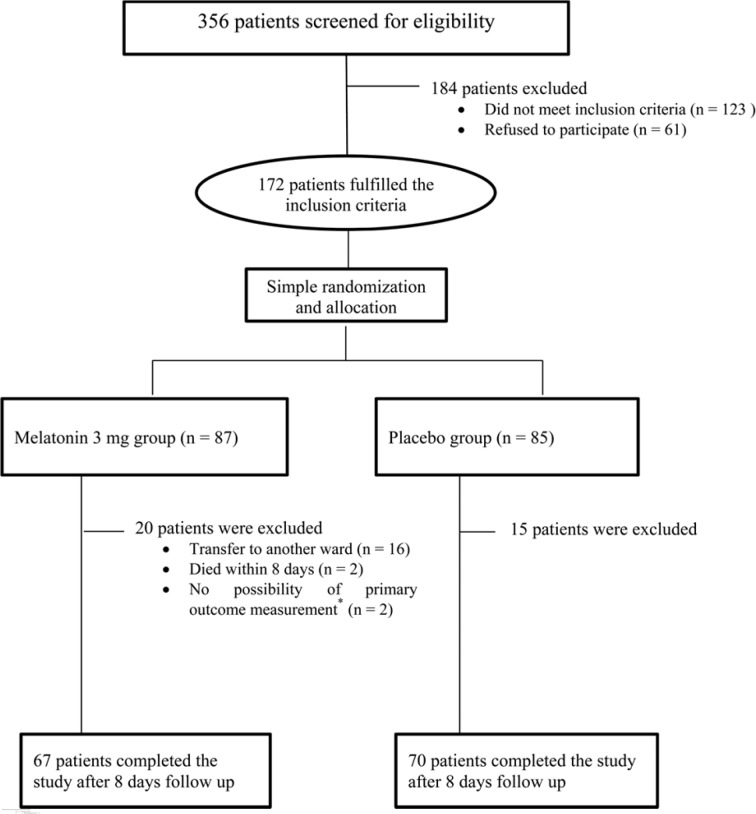
Flowchart of patients' enrolment, randomization and follow-up in different groups of the trial.^ *^Because of Glasgow Coma Score (GCS) ≤8

From October 2014 to May 2016, 172 patients among all the 356 patients admitted to medical-surgical ICU screened for eligibility within 24 h of admission met the inclusion criteria for the study. The final population of 70 patients in placebo group and 67 patients in melatonin group completed the study with the follow-up ending in May, 2016. The cases due to the transfer to another ward and the occurred mortality were the main reasons of exclusion. However, 2 patients in each group were excluded then, because delirium could not be assessed in them due to decreased GCS to less than 8 (Figure 1).

Demographic and clinical variables, which might affect delirium, were shown in Table 1. Thereafter, multiple logistic regression and linear regression were used to adjust for statistically significant factors at baseline (*p* < 0.1) between the groups. Among various parameters analyzed between groups, the reason for ICU admission (medical, surgical and trauma), prevalence of diabetes and chronic organ insufficiency, and the mean chance of delirium development during 8 days of ICU stay were significantly different between melatonin and placebo groups (Table 1). It is to note that the chance of delirium development was comparable between study groups in the medical subpopulation. However, this chance was higher, although non-significant in melatonin group within other subgroups of surgical and trauma patients (*P* = 0.08 and 0.01, respectively).

**Table 1:** Demographic and clinical data (Mean ± SD or Count (%)) of the study population

**Characteristic**	**Melatonin group** **(n = 67)**	**Placebo group** **(n = 70)**	***P*** **-Value**
Age (years), mean ± SD	52.5 ± 18.4	49.9 ± 19.0	0.46
Sex (male), n (%)	36 (53.7)	42 (60.0)	0.49
APACHE II score, mean ± SD	8.1 ± 4.3	7.3 ± 4.6	0.32
Mean SOFA score during study follow-up, mean ± SD	3.1 ± 2.0	3.2 ± 2.3	0.76
Chronic organ insufficiency*, n (%)	7 (10.4)	0 (0)	0.06
Chronic baseline diseases, n (%)			
Cardiovascular disease	26 (38.8)	17 (24.2)	0.13
Diabetes Mellitus	14 (20.9)	6 (8.6)	0.05
Neurological diseases	5 (7.5)	5 (7.1)	1.00
Respiratory disease	0 (0)	2 (4.3)	0.50
Reason of admission, n (%)			0.03
Medical	23 (34.3)	11 (15.7)	
Surgical	36 (53.7)	44 (62.9)	
Trauma	8 (11.9)	15 (21.4)	
Chronic smoking, n (%)	25 (37.3)	22 (31.4)	0.48
Chance of delirium development during 8 days of ICU stay (%), mean ± SD	8.6 ± 7.8	6.0 ± 5.1	0.02
Medical	8.3 ± 7.8	8.2 ± 7.5	0.96
Surgical	7.5 ± 7.7	5.1 ± 4.1	0.08
Trauma	13.9 ± 6.9	7.0 ± 5.3	0.01
Isolation, n (%)	31 (46.3)	37 (52.9)	0.50

SD: standard deviation; APACHE: Acute Physiology and Chronic Health Evaluation; SOFA: Sequential Organ Failure Assessment; ICU: Intensive Care Unit.

* Including congestive heart failure, liver and kidney failure.

According to Table 2, we found no significant effect of melatonin on the frequency of delirium 4.5% in melatonin versus 1.4% in placebo group, *P *= 0.36). However, it should be mentioned that melatonin effect on the frequency of delirium was partially different in the subpopulation considered in the study. Medical population is the group mostly benefitted from melatonin treatment in order to prevent delirium. 

Binary logistic regression adjusted for the reason of ICU admission, prevalence of diabetes and chronic organ insufficiency, and mean chance of delirium development did not show any significant preventive effect of melatonin on delirium (*P* = 0.80, OR = 0.71, 95% CI = 0.06-9.15).

In further analysis, we found no significant effect of melatonin on secondary outcomes (Table 2). Similarly, regression analysis after adjustments for the mentioned variables showed in our study that melatonin supplement did not significantly reduce duration of delirium (*P* = 0.86, 95% CI = -0.17-0.14, ß = -0.01), the rate of cumulative dose of prescribed haloperidol (*P* = 0.98, 95% CI = -0.22-0.28, ß = 0.04), duration of ICU stay (*P* = 0.195, 95% CI = -0.77-5.21, ß = 2.22), duration of hospital stay (*P* = 0.48, 95% CI = -3.33-6.98, ß = 1.82), and the rate of mortality (*P* = 0.92, OR = 0.94; 95% CI = 0.31-2.87).

**Table 2:** Primary and secondary outcomes reported as Mean ± SD or Count (%).

**Out come**	**Melatonin group (n = 67)**	**Placebo group (n = 70)**	***P*** **-Value**
Incidence of delirium, n (%)	3 (4.5)	1 (1.4)	0.36
Medical	0 (0)	1 (9.1)	
Surgical	3 (8.3)	0 (0)	
Trauma	0 (0)	0 (0)	
Duration of delirium (day), mean ± SD	3.0 ± 1.7	2.0 ± 1.7	0.28
Cumulative dose of prescribed haloperidol (mg), mean ± SD	4.0 ± 5.3	2.0 ± 5.3	0.32
Length of ICU stay (day), mean ± SD	8.8 ± 5.9	9.8 ± 10.6	0.50
Length of hospital stay (day), mean ± SD	18.1 ± 13.5	18.6 ± 15.6	0.85
Mortality rate during follow-up, n (%)	9 (13.4)	7 (11.4)	0.80

ICU: Intensive Care Unit.

## Discussion

This double-blind, randomized, controlled clinical trial showed no treatment effect of melatonin to decline the frequency and duration of delirium in ICU patients. Moreover, the reduced duration of ICU and hospital stay in patients receiving melatonin were not statistically different from that in the placebo group. Furthermore, we found no more significantly different secondary outcomes between groups during the follow-up. Multivariable logistic regression adjusted for baseline variables (reason of ICU admission, diabetes mellitus and chance of delirium development) also revealed similar findings. 

However, our results showed no effect of melatonin on primary or secondary outcomes, at first glance, but a more precise evaluation presented different treatment effects of melatonin in patients admitted for medical or surgical reasons. These results indicate that melatonin has more potential effects in preventing delirium in medical patients rather than the surgical ICU patients, which is consistent with the results obtained from the recent meta-analysis performed by Chen and colleagues in non-ICU setting (15).

It is possible that the imbalance of other neurotransmitters and hormones related to stress of surgical procedures such as cortisol opposed the effect of melatonin supplement in preventing delirium (21). Guo *et al.* in the recently published study declared that disturbance of both postoperative secretion rhythms of melatonin and cortisol has a significant role in developing delirium (22). On the other hand, melatonin supplement does not affect the high level of cortisol after surgery and accordingly post-operative delirium (23, 24). Moreover, the incorrect diagnosis of delirium in surgical patients linked to drowsiness of anesthesia may interfere with the results of various studies in this regard (15).

It should also be considered that the potential protective effect of melatonin supplementation for decreasing the incidence of delirium in surgical patients has been shown after hip arthroplasty in the elderly (25). It may be related to the abnormal fluctuations of endogenous melatonin levels compared with their preoperative baselines occurred in the postoperative delirium in elderly patients, and even higher incidence of delirium in patients with hip arthroplasty compared with other elderly population (15, 26).

Aging is typically associated with impairing normal circadian rhythm and could cause delirium. Previous investigations reported a significant decline of melatonin level during aging (27). Therefore, perhaps melatonin and its agonist have more pronounced effect on delirium in elderly than young population, which is shown in recently published studies regarding non-ICU settings (15). Our study had not been limited by the age of recruited patients and had relatively young population with consequently low frequency of delirium. Therefore, this relatively young age of our study population and improvement of ICU care (such as reduction in light, noise, and frequent patient orientation) are the main reasons of low frequency of delirium in our study (28). Subsequently, it is possible that the frequency of delirium in our study compared with previous conducted studies is not many in order to detect the therapeutic effect of exogenous melatonin supplementation.

Findings from previous studies that examined the beneficial effect of exogenous melatonin for sleep and delirium management in ICU patients were inconsistent (1, 29-31). Different studies showed development of delirium linked to risk factors, such as abnormal levels of melatonin and loss of circadian rhythms other than the sleep disturbance (1, 15 and 32). It should be considered that delirium assessment was not the outcome of these studies. Moreover, several factors limited the interpretation of these results. Inconsistent methods used to assess sleep improvement, small sample size of the study, imbalance of variables which could affect sleep, and different dosage and duration of exogenous melatonin administration could be addressed as the major differences of the mentioned studies (1).

Interestingly, recently randomized controlled trial conducted by Hatta *et al.* suggested that elderly critically ill patients could give protection against delirium by using a melatonin agonist, ramelteon (33). Some differences of this trial may explain the reason of its superiority to prevent delirium in comparison to previous researches and our study. The first difference is the high affinity of ramelteon compared with melatonin. It should be noted that ramelteon is 8 and 16 times more potent than melatonin for melatonin receptors “1” and “2”, respectively, and might not have an effect on endogenous melatonin secretion (15). In addition, this promising effect of ramelteon was shown in the elderly patients that may have suffered from impaired normal circadian rhythm as compared to relatively young population recruited for our study (27).

As previously stated, potential benefit of exogenous melatonin supplementation to reduce delirium incidence was also evaluated in non-ICU settings, but they also had inconsistent results due to differences in some parameters such as setting in which patients were evaluated, dosage and duration of melatonin use, and delirium diagnostic criteria (15). Although both sultan and Jonghe studies evaluated whether melatonin could decrease delirium of surgical patients, but 6 times of more dosage of melatonin was used in Jonghe study compared to that in Sultan study (3 mg versus 0.5 mg, respectively) (14, 25). Chen *et al.* concluded this high dose of exogenous melatonin could interfere with endogenous secretion of melatonin, which could explain why exogenous melatonin was successful in decreasing postoperative delirium in Sultan study, whereas it did not show any therapeutic benefit in Jonghe study. It should also be considered that these results were derived from patients with hip surgery, with high incidence of delirium, and may not be generalized to other patients with surgery that experienced lower incidence of delirium (15).

Another study performed in the medical ward evaluated low dose of melatonin (0.5 mg) for 14 days to reduce delirium incidence in elderly patients admitted for intensive care. The findings of this study represented a potential protective effect of exogenous melatonin against delirium (34). It seems that longer duration of melatonin use compared to surgical patients gives an opportunity to melatonin for modulating central dopaminergic activity in medical patients and recovery from delirium (15).

Finally, different diagnostic criteria used for delirium assessment in these studies such as abbreviated mental test (AMT) in the study by Sultan, Diagnostic and Statistical Manual of Mental Disorders, fourth edition (DSM-IV) in the study by Jonghe and confusion assessment method (CAM) in the study by Aama make it difficult to compare the results of these studies.

Although limited and inconsistent information is available regarding the dosage and duration of melatonin to prevent delirium development, but in accordance with the available findings, we suggested lower dosage (0.5 mg) and longer duration (at least 14 days) of melatonin, to reduce delirium in ICU setting for future studies. Lower dose of melatonin with less probability of disturbance in endogenous melatonin secretion and providing exogenous melatonin for longer duration to achieve the anti-inflammatory effect of that and modulation of neurotransmitter function may be effective modality to reach the potential protective effect of melatonin in delirium prevention. 

In addition, patients with high chance of delirium development such as the elderly in various subgroup of medical, surgical and trauma patients should be evaluated in future studies, in order to show which subgroup could be most benefitted from exogenous melatonin supplementation. 

Since melatonin’s plasma and urinary levels are directly related to its concentration in the central nervous system (15, 35), we recommend evaluating melatonin level in plasma or urine, during the follow-up to determine the subgroup of patients mainly benefitted from exogenous melatonin supplementation in order to prevent delirium.

Several limitations should be considered for the present study. Low frequency of delirium and significant differences in some baseline characteristics between groups are the most important limitations of our study. However, daily assessment of delirium as well as undiagnosed hypoactive delirium, especially in surgical patients, should also be considered in our limitations. Whereas, strong points of our study include the methodology with a double-blind randomized placebo controlled trial. Moreover, we used validated and reliable tools for detecting and measurement of delirium. Finally, more analysis can give a new view of the possible different role of melatonin in medical and surgical patients in ICUs.

In fact, this is a small clinical trial, and although it demonstrates primary promising results to determine which subgroup of patients could benefit from melatonin to prevent delirium and which dosage and duration of administering melatonin would be most effective for this purpose, but more studies are required in this regard.
